# Supporting respiratory patients in primary care: a qualitative insight from independent community pharmacists in London

**DOI:** 10.1186/s12913-018-3814-2

**Published:** 2019-01-05

**Authors:** Iman Hesso, Reem Kayyali, Shereen Nabhani-Gebara

**Affiliations:** School of Life Sciences, Pharmacy and Chemistry, Kingston University London, Penrhyn Road, Kingston upon Thames, KT1 2EE UK

**Keywords:** Community pharmacists, Perceptions, Technology, Respiratory patients, Inhaler technique, Adherence

## Abstract

**Background:**

Community pharmacists’ (CPs’) interventions have a positive impact on managing respiratory patients. However, methods used by CPs to assess patients’ inhaler technique and adherence are subjective. New technologies to objectively assess inhaler technique and adherence were introduced to address such a gap. This study aimed to explore CPs’ perceptions towards the management of respiratory patients regarding inhaler technique and adherence. In addition, it explored the views of CPs and their need of technologies to objectively assess inhaler technique and adherence. CPs were probed with a new technology called Inhaler Compliance Assessment (INCA) device, designed to objectively monitor both inhaler technique and adherence of patients using a dry powder inhaler, as an example.

**Methods:**

A qualitative study employing semi-structured interviews was conducted. A convenience and snowballing sampling strategy was employed to recruit CPs working in independent community pharmacies within West and South London. Twenty-three pharmacists were interviewed between August and November 2015. Data was analysed thematically using the framework methodology and coded using NVivo10 software.

**Results:**

Analysis revealed five main themes: services and limitations of patient support, the need and acceptability of new technologies to support respiratory patients, fragmented primary care, the need to promote the clinical role of CPs, and professional identity. Patient support was patchy and affected by several barriers related to pharmacists and patients. In addition, lack of communications with different healthcare professionals in primary care and inaccessibility to clinical records were identified as problematic issues. Some CPs perceived their clinical role to be lacking within the patient care pathway. Interestingly, CPs showed positive a attitude towards the use of technologies, such as the INCA technology to support patients and were willing to provide new services. However, remuneration appeared to be a major driver for willingness to offer new services or promote existing services.

**Conclusion:**

The current study highlighted some measures that can augment CPs’ clinical practice while managing patients, such as having accessibility to patients’ medical records and the use of technologies such as the INCA technology to promote objective counselling of patients.

**Electronic supplementary material:**

The online version of this article (10.1186/s12913-018-3814-2) contains supplementary material, which is available to authorized users.

## Background

**Fig. 1:**
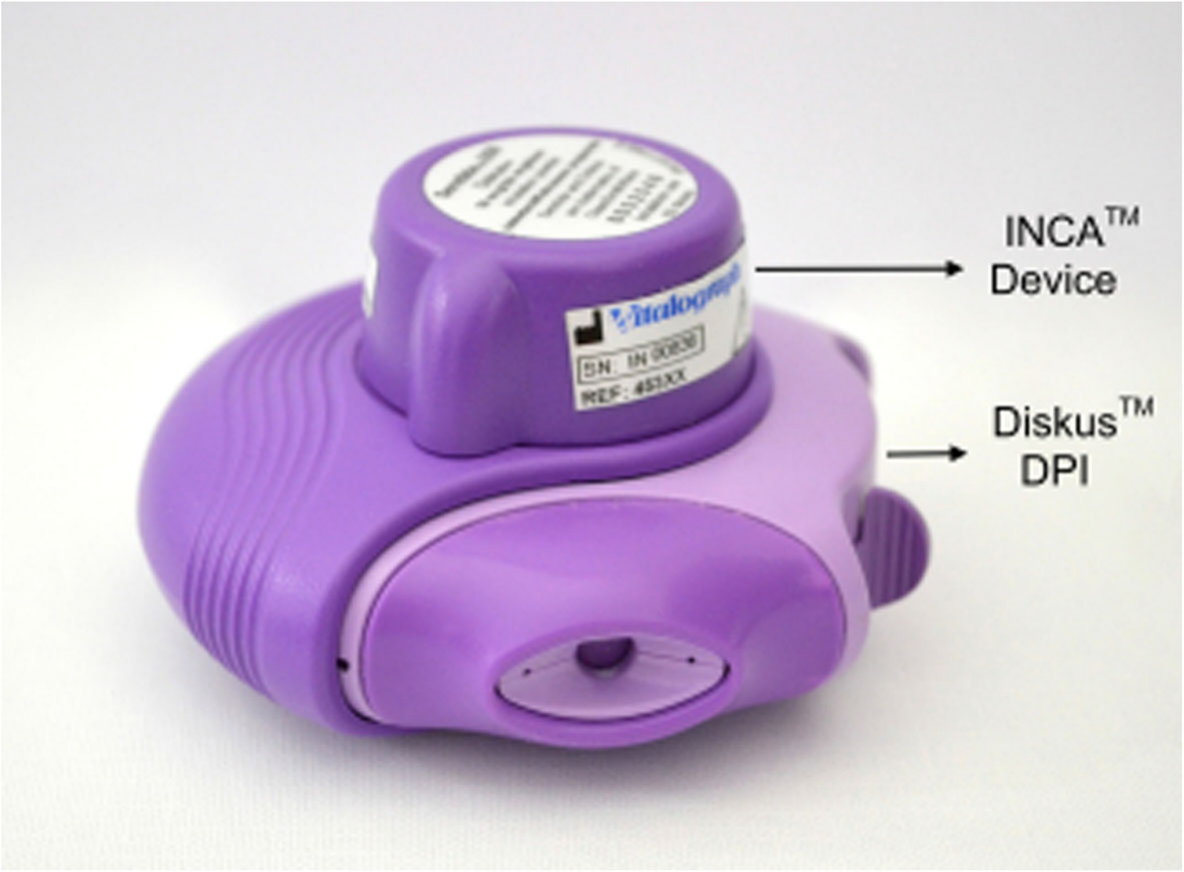
The INCA™ device attached to a Diskus dry powder inhaler (DPI) Permission to reproduce the figure of INCA™ device was granted from Vitalograph Ltd. (28)

Respiratory conditions such as asthma and chronic obstructive pulmonary disease (COPD) are examples of long-term conditions (LTCs) that have a high economic burden on the healthcare systems worldwide [1–5]. Inhaler therapy represents the backbone for the management of these respiratory conditions [1, 6]. However, poor adherence to inhaled medications and poor inhaler technique have been repeatedly identified to be problematic issues [1, 7–13]. Community pharmacists (CPs) are well placed to provide services to patients with chronic respiratory conditions [1, 12, 14–16]. In the UK, CPs offer a range of services to promote medicine optimisation in patients with LTCs, such as: the Medicine Use Review (MUR) service which is an adherence/concordance review, with respiratory patients as target group and the New Medicine Service (NMS) targeting patients who are newly prescribed a medication for a LTC [17]. Several studies highlighted the positive impact of CPs’ interventions in optimising inhaler technique and adherence for respiratory patients [18]. However, one of the limitations of the current practice is the use of subjective measures for assessing inhaler technique and adherence [10, 19, 20] . The current standard for inhaler technique assessment is a checklist method which varies between studies [1, 21] and has been reported to be subjective [22]. Strategies used to assess adherence were mainly by self-reporting (questionnaires) or using medication refill rate which in essence does not assess actual medication-taking behaviour [23]. Therefore, assistive technologies to objectively assess inhaler technique and adherence have emerged to address such a gap. However, some of these technologies assess adherence alone through recording the date and time of actuations only, such as: the Neubilizer Chronolog (NC), Smart Inhaler Tracker, SmartTrack, and SmartTouch AV [24]. Whereas, others assess inhaler technique alone, such as the Aerosol Inhalation Monitor (AIM) device [25] and Inhalation Manager (IM) [26]. One of the most recent technologies in this domain is the Inhaler Compliance Assessment (INCA^TM^) device. The INCA™ device (Fig. 1) is a novel monitoring device that has the advantage of being an automatic and objective measure of both inhaler technique and adherence at the same time while patients using the inhaler at home [19, 20, 27]. The device is manufactured by Vitalograph Ltd. [28]. It is a small acoustic, battery operated device that can be attached to the dry powder inhaler (DPI) without interfering with the mechanism of drug delivery nor the mechanics of the inhaler use [20, 22]. It provides objective feedback in the form of graphical data showing the patient’s adherence (date and time) and inhaler technique (indicating the most common error type). The feedback generated can serve as a guide for healthcare professionals (HCPs) to manage patients, especially those who are receiving an appropriate medication regime but showing no improvement in their condition [20].

The diffusion of such new technologies into practice requires certain pre-requisites and processes to ensure successful adoption and implementation. Rogers Diffusion of Innovation (DOI) [29] theory describes the process experienced by individuals or units in a social system when a new idea/innovation is presented to them. The characteristics of the innovation itself are particularly important in determining its rate of adoption. According to the theory, the following attributes are essential to facilitate the adoption of any innovation: relative advantage (the extent an innovation is perceived to be better than the current available options/practices), complexity (the degree of difficulty of an innovation in terms of understanding and usage), compatibility (the degree of consistency of an innovation with the already existing values, beliefs, experiences and needs of the individuals/adopters), trialability (the extent to which an innovation can be easily tried/experimented on a limited basis before permanent adoption), observability (the extent to which the innovation’s results/outcomes are visible to others).

This study aimed to assess CPs’ perceptions, experiences and current practice towards the management of respiratory patients with respect to inhaler technique and adherence. In addition, this study aimed to highlight CPs’ perceptions and need for an objective measure of inhaler technique and adherence, using INCA^TM^ technology as an example, given the increased recognition of the impact that technologies can have on improving patients’ outcomes when incorporated in the wider package of care for patients [30].

## Method

The research methods of this study are reported in accordance to the Consolidated Criteria for Reporting Qualitative Research (COREQ) [31] (See Additional file 1).

### Study design

A qualitative approach using semi-structured interviews was employed to address the aims of this study. The use of semi-structured interviews was deemed suitable because it allowed for in-depth exploration of ideas, views and experiences of CPs in relation to the investigated topic. This method is underpinned by phenomenology as a philosophical approach to obtain a detailed account of the phenomena under investigation from the perspectives of participants [32].

### Study setting

The study was conducted within the following boroughs in West and South London: Kingston upon Thames, Richmond upon Thames, Hounslow, Merton, and Wandsworth.

### Participants’ recruitment

A convenience sampling strategy, based on local knowledge and proximity to the researchers was used to recruit participants. A list of independent community pharmacies was identified by one researcher (IH) through conducting a search on the National Health Service (NHS) choices website for the contact details of community pharmacies located within West and South London. Multiple chain pharmacies were excluded due to research governance requirements. CPs, owning or working in independent community pharmacies within West and South London, were approached in person by the first author and provided with a written information sheet about the study to consider. The researcher then contacted the pharmacists by telephone or in person and those who agreed to participate were included and a schedule for the interview was determined. A snowballing technique [33] was also employed to identify additional CPs to interview.

In qualitative research, there is no conventional guidance to determine the sufficient sample size for interviews to be conducted [34, 35]. However, the concept of data saturation has been widely used in many studies to determine the sample size [34, 35]. Therefore, recruitment was continued until data saturation was achieved, where no new data was emerging out of the interviews. The analysis process was cyclical and iterative which involved the transcription and reading of the first few interviews to achieve familiarisation with the data collected and identify preliminary codes. After that, each interview was transcribed and coded to help guide data saturation. Data saturation was reached after the 20th interview. The stopping criterion for data saturation, which denotes the number of interviews conducted without any new data after which recruitment can be stopped, was three [36]. A total of 55 community pharmacies were approached, and 23 CPs were successfully recruited and interviewed. Most pharmacies declined participation due to either staffing or time constraints.

### Data collection

Semi-structured interviews were conducted face-to-face by the first author, a female doctoral researcher who had received training in conducting qualitative research in healthcare settings. The interviews were conducted in the private consultation room at the pharmacy to maintain confidentiality and avoid distraction. None of the research team had relationships with any of the participants prior to study commencement. Therefore, a brief introduction about the purpose of the research was provided by the interviewer prior to the interviews. Written informed consent was also acquired before conducting the interviews. Twenty-three CPs were interviewed between August and November 2015. The demographics of the interviewees are summarised in Table (1). All interviews were audio recorded and hand written notes were taken during the interviews. The interviews lasted between 20 and 35 min. No repeat interviews were conducted with any of the participants.

**Table 1:** Demographic characteristics of study participants

Demographic data, n (%)	
Gender	
Female	9 (39%)
Male	14 (61%)
Age	mean age = 42.5 years, range = 24–56 years
21–30	4 (17%)
31–40	5 (22%)
41–50	8 (35%)
51–60	6 (26%)
> 60	0 (0%)

### The interview topic guide

The interview schedule (See Additional file 2) was developed by the research team to address the aims of the study and consisted of 17 open-ended questions that covered the following sections: current practice related to inhaler technique and adherence, confidence about knowledge and services delivery, technology use within current services, and barriers to provision of support to respiratory patients. In the technology section, CPs were probed with the INCA™ device as an example, since the device is designed to objectively monitor both inhaler technique and adherence at the same time, both of which are reported in the literature to be problematic among patients [1, 9–11, 13]. The first author showed the CPs the INCA^TM^ device, a video on how to mount it to the DPI, and the graphical output generated after the analysis.

The interview schedule was piloted on 5 pharmacists working within the same academic institution of the research team and resulted in minor amendments.

### Data analysis and reporting

The audio recorded interviews were transcribed verbatim, anonymised and analysed by the researcher who conducted the interviews. Transcripts were not returned to participants for comments. Data analysis was done thematically by the first author based on the five-stage framework approach [37–39]. The first stage involved listening to the recorded data and reading and re-reading a small number of interview transcripts and any written notes to become familiar with the data. The patterns within the responses in relation to CPs' experience of managing respiratory patients and the obstacles they perceive to affect their role, together with their experiences in using technology and its need to provide objective monitoring of inhaler technique and adherence, and the participants’ use of language while describing their experiences were examined. This enabled the identification of the initial emergent codes.

The second stage involved the formation of the thematic framework to structure the analysis. The coding framework was developed through a recursive process, which was agreed by all researchers and involved grouping of the initial codes and a priori literature review into categories, which then formed the working analytical framework. The broad categories identified were: patients’ management and technology use. The remaining transcripts were read and re-read to ensure that all data had been coded and the analytical framework was further developed as additional codes emerged. Codes were then examined and grouped into meaningful themes and subthemes where applicable. Hence, derivation of themes was done using inductive/deductive approaches. All themes were given an equal weighting within the thematic framework. Numerical Indexing was done to the developed framework by assigning numbers to emergent themes and associated subthemes, which was then applied to the transcribed data. Charts were then created, which summarised the respondents’ views and experiences within the emergent themes and subthemes . This was followed by mapping and interpretation of the data in relation to the research objectives [37].

The transcripts were managed and coded using NVivo10 software. The coded transcripts were continually and extensively discussed, and checked by the other co-authors RK and SNG, which at times involved modification of the emergent themes. RK has a Doctor of Philosophy (PhD) qualification and SNG has a Doctor of Pharmacy (PharmD) qualification and both are female university academics who have considerable experience in conducting qualitative research. Agreement about all the emerging themes and subthemes was verified by all authors to ensure validity and consistency of the findings and to overcome bias in data coding.

The results are presented in the form of themes and subthemes. Participants’ quotes are used to substantiate the findings presented under each theme. The theme regarding technology acceptability was interpreted using the Rogers DOI theory [29].

### Ethical consideration

The study received ethical approval from the Research Ethics Committee at Kingston University London (Reference No. 1415/034).

## Results

Analysis of the interviews revealed five main themes (Table 2). Additional quotations to those presented under each theme are available in Table 3.

**Table 2:** Emergent themes and subthemes

Theme	Subtheme (s)
Services and limitations of patient support	Services provided to respiratory patients Barriers to patient support
Fragmented primary care	Lack of communication with other HCPs Lack of access to patients’ records
The need and acceptability of new technologies to support respiratory patients	Relative advantage Compatibility Complexity Trialability and observability
The need to promote the clinical role of community pharmacists	
Professional identity

**Table 3:** additional quotations from respondents

Theme/ Subtheme	Respondents’ Quotations
Theme 1: Services and Limitations of patient support	
Subtheme: Services provided to respiratory patients	*“We do MURs or NMS for these patients…. We also do flu vaccination and smoking cessation.”* (CP10) *“MURs, we are targeting respiratory MURs, smoking cessation my colleague does…. We do NMS… We do flu vaccines at the moment”* (CP13) *“Usually NMS and Smoking cessation service, MUR, flu vaccination.”* (CP14)
Subtheme: Barriers to patient support	*“I think biggest barrier would be time, resources. It is basically, I think we need to think about rearranging our time table so it would mean that we would have to have a little bit of extra resources, the only time I bring people in the consultation room when I have a locum”. (CP6)* *“Lack of training probably and again yea not having placebo inhalers, these two things” (CP7)* *Patients’ attitudes and cooperation to have the service, the ability to recruit patients, the willingness to participate, and the time we have to spend with patients (CP16).* *“Time, because it can take 10–15 min each intervention especially when you want to check technique. Correct reimbursement, because it comes out of our time. Barriers would be mainly time and reimbursement. If we got to have tools supplied on a regular basis like my placebos go back to while ago so I have to keep asking the reps, we do not see the reps any more so we don’t get placebos unless we ring the manufacturer.”* (CP10)
Theme 2: Fragmented primary care	
Subtheme: Lack of communication with other HCPs	*“And there is also I feel lack of information coming from the prescriber, for example: with the preventer steroid inhalers they don’t say to them (the patients) use it regularly… so they (the patients) just use the reliever whenever they need, and with the other one (preventer) they end up in trouble because they don’t realise it is far better to manage.”* (CP11) *“There is a bit of resistance from patients because they are saying we are repeating what the practice nurses may have done. The practice nurse may have gone through all this with them and then they are wondering why is the pharmacist doing it as well”.*(CP17) *“…even we can’t access the GP surgery, communication is so bad, telephones are always busy, receptionists get the doctor to call back…”* (CP3)
Subtheme: Lack of access to patients’ records	*“I think what is a barrier at the moment and what would help us and I think it is for the future, they are going to allow us clinical access with our smart card for us to know so with our smart card not only we will be able to look at the drug history of the patient but we should be able to look at the clinical history.”* (CP4)
Theme 3: The need and acceptability of new technologies to support respiratory patients	
Subtheme: Relative advantage	*“it shows they are using their device properly, using it on timely basis because unlike when I am talking to them, I am trusting them to say yes I am using my inhaler. I am depending on the trust; I am using my inhaler twice a day without fail. Whereas, this device would say you don’t use your device twice a day, you are telling me that you use it. It allows us to actually say here you are to proof that sometimes you are not using the device properly, sometimes you are not taking enough drug, sometimes you are missing it totally, you are not using it enough etc…so yea I am for that technology”* (CP12) *“yea definitely, I think it will be good for some patients because at least this way even doctors will be able to get feedback about this. The information you are getting/creating here gets referred back essentially to a prescribing body about usage and about the technique being used correctly. There is no point of prescribing something and then not assessing the patients, there is a gap between usage and compliance and being used properly. I mean you need to make sure the drug is being used effectively so then assess the patient thereafter so this information should be good for the doctor to get back.”*(CP18)
Subtheme: Compatibility	*“Sometimes it is easier with technology, isn’t it? It can make things easy for you.”* (CP10) *“We see the information gathering from technology tends to be more useful so information gathering in old ways tends to be difficult to actually utilise whereas it tends to be easier analysed with that technology to benefit the patient. Easier to analyse and use.”* (CP13)
Subtheme: Complexity	*“I mean as long as it is efficient, as long as it does not affect therapy then I will be happy to and as long as it is easy to use.”* (CP14) *“It has some beneficial factor (2b) and easy to use (2c). Going with the time. Sometimes it is easier, isn’t it? It can make things easy for you.”* (CP10)
Subtheme: Trialability and observability	*“By collecting preliminary data, if something new or new sort of advice come up that would bring benefit to the patient so obviously yes.”* (CP15) *“If I am offering something that I tried and I know works, they (patients) are advised that it works and if they use it and find it works then they are more likely to be happy.”* (CP11)
Theme 4: The need to promote the clinical role of community pharmacists	
	*“In MUR we will find out whether or not the product is working, if we see that the product is not actually doing what is supposed to do then we have to refer them back to the GP, it is not for us to say, we can’t make clinical recommendations.”* (CP16)
Theme 5: Professional identity	
	*“We would like to have the funded COPD service back when we were actually caring for the patients before when we were providing the COPD service….But you have to value the pharmacist time then, at the moment nobody is valuing the pharmacist time. If I was providing all these services and not getting paid for it then there is no value to my service yea, there is no value to my establishment, where I am going to get the money for to provide all this, right?” (CP3)* *“You can spend 10 min with the patient but you can’t spend half an hour unless it is properly funded you know that’s the only thing, it is easier to spend 10 mins that’s fine but if you are going into a lot of details with the patient then you need an extra funding to do all the extra services. It’s like an extra service, then we can spend half an hour with the patient and go through all the queries and all the problems.”* (CP21)

### Services and limitations of patient support

#### Services provided to respiratory patients

All CPs highlighted that they provide support to respiratory patients through services within the community pharmacy contractual framework, mainly: dispensing, repeat dispensing, MUR, NMS, flu vaccination and smoking cessation. Supporting patients with inhaler technique and adherence was mainly done during the MUR service which is conducted once yearly if the patient accepts to have the service, or opportunistically over the counter if the patient raised a query.

*“We do respiratory MURs as part of targeted MUR group, we do NMS enhanced service for new patients starting on a different inhaler whether asthma or COPD, we also do flu vaccination and smoking cessation.”* (CP19).

#### Barriers to patient support

Several barriers were reported by CPs to affect support and service provision to respiratory patients. Ten CPs indicated providing MURs to respiratory patients every two or three years and not on annual basis, because it was difficult to get the same patient to do the MUR each year, despite the fact that respiratory patients are a target group for this service.

*“MUR is an annual thing but it all depends that if we get the patient again the following year so they may have one off or it might be every two or three years, it just depends on the catchment.”* (CP12)

*“…even just for the yearly MURs people are not willing to take part and that is only once a year”* (CP3)

Other barriers for services provision included: pharmacists’ time and workload, lack of training, lack of incentives/re-imbursement, financial barriers, lack of resources (placebo inhalers, manpower), patients’ time in addition to patients’ health beliefs and attitudes.

*“It is basically more about time, time regarding you are busy dispensing medication, you do not have time unless there is another pharmacist working at the same time.”* (CP4)

*“A lot of people don’t want to do it (the MUR service), not every year. Like I said before people’s attitudes and people’s adherence is human nature, their beliefs.”* (CP3)

Therefore, patient support is patchy and opportunistic depending on several factors related to either patients or pharmacists.

### Fragmented primary care

#### Lack of communication with other HCPs

The need and importance of collaboration between all HCPs to promote care for respiratory patients was clearly portrayed in CPs’ responses. Fragmented care and lack of communication between HCPs were identified as main concern. According to CPs, patients currently tend to get services and information from different HCPs without synchronisation and consistency; this can lead to confusion among patients. The fragmented communication between HCPs means that CPs had no clear idea about services offered to patients and, therefore, their level of knowledge.

*“I think the other thing which could be viewed as a problem is the multiple approach from different parts of the NHS …so you are having different things which are not talking to each other...*” (CP13)

One CP even highlighted their unsuccessful attempt to promote a clear referral pathway between their service and the general practitioners (GPs).

*“You know I tried the surgeries, I spoke to all the nurses and GPs and said if any patient comes to you, you can get them come to me. I am quite happy I am not going to charge anything and I will refer anybody up to you if there are any issues. No one.”* (CP11)

The use of the phrase ‘I am quite happy’ denoted that this respondent was very keen to promote the management of respiratory patients through such initiative, yet their statement also reflected the extent of fragmentation in primary care.

#### Lack of access to patients’ records

Furthermore, lack of access to patients’ clinical history represented a challenge to provision of services, especially for new patients.

*“If you are coming to me first time I know nothing about you but if I had access to your clinical history….it is easier for us to help you...”* (CP3)

### The need and acceptability of new technologies to support respiratory patients

More than half of the interviewees perceived the adherence level of their patients to be poor. Assessing adherence level during consultations was reported to be challenging. Eight CPs reported that they had to take the patients’ word for it, while others (*n* = 9) assessed this by looking through the patient medical records (PMRs) to see the frequency of collecting inhalers as an indicator of adherence.

*“…it is difficult to know really. Obviously you can go through it from the PMR and you can see the frequency of dispensing....”* (CP17)

*“….you ask them about their adherence but what they tell you whether it is true or not you do not know. Most of the time they say they are pretty good, I mean they admit one odd missed dose but usually they say they take them regularly*.” (CP1)

*“… just from what they say and to see how often I get prescriptions for the blue inhaler, if I know it has been over prescribed then they are not using their preventer often, and I just ask, there is no other real way to assess adherence”.* (CP9)

Patients’ inhaler technique was also reported to be problematic by six CPs, who highlighted that there are always errors in inhaler technique upon checking with patients.

*“…. each MUR we find there is something in the technique that needs correction….”* (CP6)

*“….there are a lot of patients they can’t use these turbo devices because it is just too difficult for them to make the device work, whereas, the easier is the press device but then likewise you get dexterity problems. Older people can’t actually synchronise, they can’t press”.* (CP12)

*“…there are quite a lot who make mistakes and who don’t really know what they are doing which is why I think they are targeting them as MUR subjects.”* (CP13)

Therefore, when INCA^TM^ technology was discussed as an example with the interviewees, they were receptive and open to the idea. The interviews highlighted no pre-use of technology apart from YouTube videos for education.

*“During MURs, I advise them to watch some videos on Youtube. Otherwise, nothing related to technology”* (CP11)

*“No tools related to technology.”* (CP15)

The analysis revealed certain pre-requisites as essential for adopting any technological innovation, such as the INCA^TM^ device. The five attributes of innovation mentioned by DOI theory [29] were depicted in the CPs’ responses.

#### Relative advantage

The benefits perceived were related to the advantage of the INCA^TM^ device in providing objective evidence about the patient’s inhaler technique and adherence, instead of trying to get this information from the patient, which may not necessarily be accurate. The INCA^TM^ technology was perceived as an easier way to monitor patients and assess adherence, thus providing better and more accurate service.

*“It will be a bonus definitely, because if you can get the results on adherence and technique…... Having the results of that particular piece telling me that the evidence level is this, then I can investigate why, whereas now in the consultation room I will be spending time to find out about that adherence level and still not get it right whereas with this I have evidence.”* (CP19)

#### Compatibility

The INCA^TM^ technology was perceived to be compatible with the needs of CPs for novel ways that promotes patients’ understanding about inhaler technique and adherence.

*“If it makes it easier for patients to understand, then why not.”* (CP 9)

However, the technology needs to be compatible with time pressure and funding available for CPs.

*“I think it is the way forward where we should be developing the services and the advice we give and be paid for it…..Well any new service you do you need to be paid for it, you can’t just do it for free. It’s time, isn’t it?”* (CP20)

#### Complexity

CPs were willing to incorporate the INCA^TM^ technology while supporting respiratory patients as long as it was deemed to be safe and easy to use and not time consuming.

*“As long-as it is easy to use, so patient friendly…”* (CP13)

Pharmacists proposed the need for proper training to ensure ease of use.

*“Yes, I will do it, but we need training”* (CP5)

*“Yea, as long as there is training, I like technology; I have an interest in it.”* (CP11)

#### Trialability and observability

Some CPs (*n* = 4) commented on the necessity of trying the new technology on a few patients and if tangible benefits were observed in terms of patients’ outcomes, then they can apply it to the rest of the patients, which in this case refers to the trialability and observability attributes of an innovation.

*“There is no harm in trying, if after at the first 10-15 patients does not seem there is any benefit then we can always revert back to just old kind of normal way ….”* (CP9)

This highlights the positive attitude among the interviewed CPs towards the proposed technology. The responses were also reflective of the prerequisites described in Rogers DOI theory [29] to facilitate the adoption of an innovation.

### The need to promote the clinical role of community pharmacists

MUR service was found to be the main form of support to respiratory patients. Interestingly, four CPs perceived that MUR was mostly about checking medication usage with no clinical input in it. One attributing the clinical input to be only part of the full medication review which is a locally commissioned service rather than the MUR service.

*“You check how they use their medication that’s all, there is no clinical input or anything else, and it’s just the usage, it is not review really”.* (CP1)

Three CPs did not feel that a clinical role within MUR provision is within their remit, while others (*n* = 3) highlighted the need to develop such a clinical role to improve patients’ outcomes.

*“Yes (referring to discussing the condition treatment during MUR), but we can’t get involved clinically because it is not our remit that’s down to the surgery…”* (CP12)

*“I think when we get it (the services) right, it definitely improves the patient’s view of us as professionals…We do have quite a lot of patient contact but we want to make that a bit more clinical if we can….”* (CP13)

The above quotes are particularly noteworthy since they raise the question as to how CPs perceive their role; particularly, the clinical aspects associated with this role.

Interestingly, the CP quoted below commented that many of the tasks that are currently done by the CP, such as inhaler technique or flu vaccination, can be done by support staff if they are properly trained to back-up the pharmacist. This was perceived as a way to create a space/time for the CPs to develop their clinical role, which was in their view restricted to stepping up and down the treatment.

*“There is a lot of stuff that can be done by support staff, it does not have to be a pharmacist…… Is it inhaler technique? Do you need a pharmacist to check the inhaler technique or can use one of your support staff, where the pharmacist has to intervene is when you are looking at therapy and it is not working and then either increase or decrease, it is the clinical aspect we need to develop, our clinical role better. Flu jabs, why do you need a pharmacist to do flu jab? Why can’t support staff do it? If they are appropriately trained…”* (CP20)

Therefore, the perceived understanding of the CPs to their role can impact the quality of services offered to patients and their perceptions about the services.

### Professional identity

An emerging theme from the data was related to CPs’ professional identity. Five CPs believed that the proper management of respiratory patients, especially COPD, is a neglected area. They perceived that this necessitates having a separate service with a more dedicated time, due to the high economic burden of COPD on the NHS. Analysis revealed how CPs’ willingness to provide more services was conditioned by remuneration, despite the perceived need to promote the management of respiratory patients, especially high-risk ones. Nevertheless, the conditioned remuneration was justified by three CPs in the light of the potential benefits in reducing hospital admissions.

*“…if there are any ways of making this sort of thing “COPD management” a part of mainstream community pharmacy services that will be a lot easier ….This is more of a sort of an administrative and reimbursement…”* (CP23)

*“I am sure the government will save money if they included that in one of our services we get reimbursed for…The money they save from hospital admissions will be well worth it. I mean how many COPD patients keep going into hospital and if we keep an eye on them but get paid for it I will make the effort to, you know, staying on the top of their treatments. Incentives, I guess are always going to be financial in some ways, isn’t it?”* (CP10)

Interestingly, CPs were open to even upgrading the current services to include INCA^TM^ technology if remuneration is available similar to services offered by other HCPs.

*“…we should have remuneration for it not just as part of the service. GPs get paid for just measuring blood pressure which should be really the job anyway…so it is things like this, that mentality has come through now to the pharmacists: if there is no money involved they do not want to do, remuneration.”* (CP8)

*“If we get paid for it. It is brilliant. I would say it is an enhanced service so if you want me to do that you got to pay me to recruit the patients and also monitor the patient, make sure they are compliant etc.…. it is got to be some sort of incentives. At the moment you can just give the inhaler out but if you want to see improvements and see if they are compliant and you want me to put a device on it (the inhaler) then I need to monitor and I have to call the patient in.”* (CP20)

## Discussion

The findings generated in this study shed the light on several issues that were found to be challenging and problematic in terms of managing respiratory patients in the community. Several barriers have been identified; some of these were related to CPs, such as time, workload, lack of resources, lack of incentives, while others were related to patients, such as patients’ health beliefs, patients’ attitudes and lack of time. These are similar to the barriers cited by CPs while supporting asthma patients in Australia [40].

Fragmented care and lack of communication among HCPs in primary care were perceived by CPs to be negatively affecting care provision. This echoes the findings of previous research which showed that fragmented care and lack of communication among HCPs were perceived as a challenge among COPD patients, informal carers and HCPs in four European countries [41, 42]. This is further escalated by the fact that there is no clear role for the CP in the COPD patient care pathway [43]. In another research focusing on CPs’ experiences as service providers in an implementation trial of a specialist asthma service in Australia, CPs have identified collaboration with GPs as a key challenge [44]. Poor integration with other parts of the NHS has been identified in a recent review by NHS England to be among the barriers to better utilisation of the community pharmacy workforce [45]. This, in return, stresses the importance of tackling the problem of poor integration and communication between HCPs, as a way to enhancing care provided to patients.

In the current study, CPs did not clearly recognise their clinical role within the MUR, and perceived a need to promote this role, which highlights lack of recognition of inhaler technique as a clinical intervention among CPs. For some CPs, the clinical role was restricted to stepping treatment up and down for patients. However, for respiratory patients, having correct inhaler technique is pivotal for optimal drug delivery to the lungs [46]. Errors in inhaler technique result in no drug or a reduced amount of drug reaching the lungs [21, 47]. Consequently, rectifying inhaler technique is a purely clinical aspect which can be done by the CP in an MUR. Therefore, this raises an important question about the CPs’ negative perception of the extent of their clinical role. A previous research highlighted that hospital pharmacists identified themselves more with the clinical practitioner identity as compared to CPs [48]. Hospital pharmacists defined clinical work as that undertaken at the patient’s level which involves applying knowledge about medicines to a person’s condition. Even though CPs described their role as spending time with patients talking about symptoms and treatment, they did not explicitly refer to it as being ‘clinical’ [48].

CPs do not usually have full access to the clinical history of the patient and the Summary Care Record (SCR) accessible to UK CPs may not contain adequate information [45, 49]. This was highlighted as a limitation to effective service delivery for respiratory patients. The importance of having full access to clinical information has been previously emphasised [45, 50–52]. Similarly, in a study interviewing CPs about their role in dementia care in the UK, CPs echoed the lack of clinical role in this area and attributed that to isolation, lack of access to patients’ clinical records, poor integration into the healthcare system and being viewed as shopkeepers which provided a sense of inferiority [49]. This in return provides a supportive explanation as to why CPs perceived their clinical role to be lacking, since fragmentation of care and lack of access to patients’ clinical records were raised as problematic issues. Furthermore, a previous research in Scotland indicated that patients with LTCs had more trust in the GP regarding their disease management, due to their access to the full medical history, and were less likely to approach CPs, as the services offered were perceived to be incomplete [53]. This in return highlights the importance of granting CPs’ access to the clinical records of patients. In fact, the rollout of SCR into community pharmacies started in England in mid-2015 and was expected to be completed by end 2017 [54, 55]. Despite that SCR do not include enough information as highlighted earlier, yet CPs should recognise this initiative as a potential opportunity to promote the way they engage with patients and promote the clinical aspects of the services they deliver. The recent update as per May 2018 indicates that more than 93% of community pharmacies have access to SCR and more than 24,000 pharmacy professionals completed their SCR e-learning [56]. Another approach which would also enable CPs to embrace a greater clinical role [57–59] would be pharmacy independent prescribing, which has been introduced in the UK since 2006 [58, 60]. However, the implementation of pharmacist prescribing is poor in community pharmacy setting compared to other sectors, such as hospital and primary care (GP surgeries) [57]. Whilst some barriers to independent prescribing are common across all pharmacy sectors, CPs face additional challenges [57, 58]. In the current study, none of the respondents have reported to be qualified as an independent prescriber.

Poor adherence to inhaled therapy is directly associated with poor clinical outcomes and increased healthcare expenditure [13, 61, 62]. In fact, most participants indicated that patients’ inhaler technique and adherence were a challenge. This is well documented in the literature [9, 11, 13, 18]. Responses of CPs reflected positive attitudes towards the INCA^TM^ technology and demonstrated the need for such a technology as illustrated by Rogers DOI theory [29] in terms of the attributes that are essential for the adoption of new innovations. The INCA^TM^ technology was perceived by CPs as an educational tool [20] that provides objective feedback on patients’ inhaler technique and adherence level in real-life situations, compared to the subjective measures used currently [10, 19, 20]. This technology can help CPs in providing personalised advice that is tailored to patients’ needs during consultations, which can support the notion of a clinical role within the MUR. Thus, the introduction of assistive technologies such as the INCA^TM^ technology can be of particular potential, given the poor engagement and adherence to treatment reported among respiratory patients in the literature [13]. The provision of tailored education using quantitative feedback has been associated with a higher magnitude of improvement in comparison to the current best practice education [63].

Despite perceiving and advocating such potential opportunities, yet CPs willingness to provide new services or upgrade existing services with technology was mainly driven by finances (proper funding and correct re-imbursement). Pertaining to this comes the dual professional/commercial role of CPs, which has been a subject of continual discussion [64] and their professional identity. The study of Elvey et al. [48] showed that while pharmacists were trying to distance themselves from being viewed as shopkeepers, being a business person was an important part of the identity due to the satisfaction attained by having their own business and autonomy. It is clear that although GP and pharmacy services are commissioned in a similar fashion in the UK, the work setting adds to the business perception of pharmacy. Two qualitative studies in the UK [65, 66] reported how the professional identity of CPs underpinned many of the perceived barriers to provision of services to patients with LTCs, including lack of remuneration and the retail environment of community pharmacy.

### Strengths and limitations

The current study provided an in-depth account into CPs’ perceptions about current care and technology use for supporting respiratory patients. To our knowledge, few studies exist about CPs’ perceptions regarding the use of technology for supporting patients with inhaler technique and adherence. Data saturation was ensured during data collection and all interviews were conducted face-to-face. Despite of the fact that the CPs were randomly approached, the selection bias might have occurred, since participation is more likely to involve the more motivated CPs or those with more interest in respiratory conditions. Another limitation of the study is that perceptions were only explored among CPs owning or working in independent community pharmacies, thus limiting the generalisability of the results across the English community pharmacy sector.

## Conclusion

The current study highlighted some factors affecting the management of respiratory patients in community pharmacies in London, such as fragmentation of primary care and perceived lack of clinical role. Issues pertaining to professional identity, notably remuneration, appeared to be a major driver for willingness to offer new services or promote existing services. The study also highlighted some measures that can augment CPs’ practice while managing respiratory patients, such as the use of technologies, for example the INCA^TM^ technology, to promote objective counselling to patients regarding inhaler technique and adherence. The study also highlights the need for clinical role recognition and facilitation through accessibility to patients’ records to enable CPs to have a more recognised role in respiratory patients’ care pathway.

## Additional files

Additional file 1: COREQ checklist. (DOCX 20 kb)

Additional file 2: The interview schedule. (DOCX 17 kb)

## Abbreviations

AIM: Aerosol Inhalation Monitor
COPD: Chronic Obstructive Pulmonary Disease
COREQ: Consolidated Criteria for Reporting Qualitative Research
CPs: Community Pharmacists
DOI: Diffusion of Innovation
DPI: Dry Powder Inhaler
GP: General Practitioner
HCPs: Healthcare Professionals
IM: Inhalation Manager
INCA: Inhaler Compliance Assessment
LTCs: Long-term conditions
MUR: Medicine Use Review
NC: Neubilizer Chronolog
NHS: National Health Service
NMS: New Medicine Service
PharmD: Doctor of Pharmacy
PhD: Doctor of Philosophy
PMR: Patient Medical Record
SCR: Summary Care Record
