# Supplementary material for: Supporting respiratory patients in primary care: a qualitative insight from independent community pharmacists in London
Source: BMC Health Serv Res. 2019 Jan 5;19:5. doi: 10.1186/s12913-018-3814-2 (PMC6321650; doi:10.1186/s12913-018-3814-2)
Supplement: Supplementary file 1 — COREQ checklist. (DOCX 20 kb) [file 12913_2018_3814_MOESM1_ESM.docx]

Consolidated Criteria for Reporting Qualitative Research (COREQ) checklist:

| COREQ Criteria | Criteria fulfilment in the current research |
| --- | --- |
| **Domain 1: Research team and reflexivity** | |
| **Personal Characteristics** | |
| 1. Interviewer/ Facilitator | The interviews were conducted by the first author and this was mentioned in the methodology section (lines 209). |
| 2. Credentials | Credentials of the research team were mentioned in the methodology section (lines 210, 271-272). |
| 3. Occupation | Occupations of the research team were mentioned in the methodology section (lines 210, 272-273). |
| 4. Gender | The research team consisted of three female academics and this was mentioned in the methodology section (lines 210, 272). |
| 5. Experience and training | The research team have considerable experience in conducting qualitative research in healthcare settings and this was mentioned in the methodology section (lines 210-211, 273-274). |
| **Relationship with participants** | |
| 6. Relationship established | None of the research team had relationships with any of the participants prior to study commencement (lines 213-214). |
| 7. Participant knowledge of the interviewer | There was no prior knowledge between the interviewer and the participants, that’s why a brief introduction was provided by the interviewer which stated the reasons for doing the research. Furthermore, a participant information sheet was provided to all interviewees about the reasons and goal of the research (lines 192-193, 214-216). |
| 8. Interviewer characteristics | The only reported characteristics about the interviewer is the qualification (line 210) and that the interviewer had no previous relationships with the interviewees (lines 213-214). |
| **Domain 2: Study Design** | |
| **Theoretical Framework** | |
| 9. Methodological orientation and theory | The main methodological orientation was phenomenology (line 176). However, framework approach was used for data analysis (line 240). |
| **Participant selection** | |
| 10. Sampling | Convenience and snowballing sampling techniques were used to recruit participants and this was reported in the methodology section (lines 184, 195-196). |
| 11. Method of approach | Participants were approached in person and also contacted by telephone or in person afterwards (lines 191-195). |
| 12. Sample size | 23 community pharmacists were interviewed for this research. Sample size was determined by data saturation and a stopping criterion of 3 interviews, this was mentioned in the methodology section (lines 205-206, 217-218). |
| 13. Non-participation | 32 community pharmacists refused to participate. Reasons for declining to participate in the interviews were provided in the methodology section (lines 206-207). |
| **Setting** | |
| 14. Setting of data collection | The interviews were conducted in the workplace, i.e.: private consultation room in the pharmacies (lines 211-213). |
| 15. Presence of non-participants | The interviews were conducted in the private consultation room to maintain confidentiality and avoid the presence of non-participants during interviews (lines 211-213). |
| 16. Description of sample | Basic characteristics about the interviewees such as age range and gender were provided in the methodology section (lines 218-219). |
| **Data Collection** | |
| 17. Interview guide | A copy of the interview guide was provided as an additional file. |
| 18. Repeat interviews | No repeat interviews were conducted and this was mentioned in the methodology section (lines 221-222). |
| 19. Audio/ visual recording | All interviews were audio recorded (line 219). |
| 20. Field notes | Hand written notes were taken during the interviews (lines 219-220). |
| 21. Duration | Duration of the interviews was mentioned in the methodology section (lines 220-221). |
| 22. Data saturation | Sample size was guided by data saturation and this was reported in the methodology section (lines 199-205). |
| 23. Transcripts returned | Transcripts were not returned for participants to check and comment (lines 238-239). |
| **Domain 3: analysis and findings** | |
| **Data analysis** | |
| 24. Numbers of data coders | Data coding was done by the first author. However, the coding framework was extensively discussed and agreed by all authors (lines: 240, 251-254, 269-270, 274-276) |
| 25. Description of the coding tree | Broad categories which guided coding were mentioned in the methodology section. Description of the coding tree was provided, please see lines 240-267 in the methodology section. |
| 26. Derivation of themes | Derivation of themes was done using inductive/ deductive approaches i.e.: from the data and literature review (lines 251-253, 259-260) |
| 27. Software | Management and coding of the data was done using NVivo 10 software (line 268). |
| 28. Participant checking | This was not conducted. |
| **Reporting** | |
| 29. Quotations presented | Participants’ quotations were included in the results section, and this was mentioned in the methodology section (line 278). |
| 30. Data and findings consistent | The final themes and subthemes were checked and verified by all authors to ensure validity of interpretations and consistency of the findings (lines 274-276). |
| 31. Clarity of major themes | All themes were given an equal weighting within the thematic framework (line 260-261). |
| 32. Clarity of minor themes | All themes were given an equal weighting within the thematic framework (line 260-261). |
