# Supplementary material for: Supporting respiratory patients in primary care: a qualitative insight from independent community pharmacists in London
Source: BMC Health Serv Res. 2019 Jan 5;19:5. doi: 10.1186/s12913-018-3814-2 (PMC6321650; doi:10.1186/s12913-018-3814-2)
Supplement: Supplementary file 2 — The interview schedule. (DOCX 17 kb) [file 12913_2018_3814_MOESM2_ESM.docx]

## Appendix (1): interview schedule:

1. **Managing / supporting patients with asthma and COPD**
2. Can you provide me with an overview of the services that you offer to asthma and COPD patients in your pharmacy?
3. What are the common interventions that you offer to asthma and COPD patients during MUR/NMS? (e.g.: treatment optimisation (stepping up or down); life style advice; inhalation advice etc….)
4. What kind of information do you usually offer for asthma and COPD patients? How do you provide this information and advice? (Verbal, written, or other forms….)
5. How do you perceive the adherence level among your asthma and COPD patients? What do you think are the barriers for promoting high level of adherence among this category of patients?
6. How do you support asthma and COPD patients to understand more about their condition and adherence to their medication based on the services you mentioned?
   1. Prompt: can you please explain with example(s)/ please use examples to illustrate this.
7. How do you support asthma and COPD patients who are using inhalers with the inhalation technique based on the services you mentioned?
   1. Prompt: can you please explain with examples/ please use examples to illustrate this.
   2. Prompt: Do you use certain tools and devices (e.g.: placebo devices, inhaler training devices like In-check Dials)
   3. How frequently do you offer advice about inhalation technique to asthma and COPD patients?
8. **Confidence about service delivery and knowledge**
9. How confident do you feel about the knowledge you have concerning asthma and COPD management? Why?
10. How confident do you feel about delivering services such as MUR, smoking cessation, and vaccination to asthma and COPD patients? Why?

Prompt: Do you have the same level of confidence about all the interventions you perform within these services?

1. How do you assess knowledge gained by your patients after delivering a service?

10. What educational material, if any, do you provide to patients to take home? (For example: if they provide information about Inhaler Technique, disease and symptoms and if they provide these information for patients to take home)

11. What type of support (e.g.: training, education) have you received to deliver these services in your pharmacy?

Prompt: for training or education: how often do you receive training/education

12. How do you feel these services add to your role as a community pharmacist? And how do you perceive patients’ acceptance to the offered services in your pharmacy.

**C) Use of Information Technology (IT) within service delivery**

13. What tools do you use when counselling or conducting services like MUR or NMS for asthma and COPD patients? (Tools such as leaflets, YouTube videos, mobile applications, guidelines etc….)

Prompt: Do you have any that are related to technology or technology based?

14. How confident do you feel about delivering a service involving Information Technology (IT) e.g.: mobile application, digital decision system to support your patients?

15. Will you be opt to deliver a technology based service for your patients in the future (like INCA device technology for example)? Why?

16. In which way would that be beneficial to your patients, your role and the services offered?

N.B.: To get perceptions regarding INCA technology, the researcher will show community pharmacists the INCA device, a video on how to mount it to Seretide Accuhaler, the instructions of usage and the graphical output generated after analysis of the sound files.

**D) Barriers to providing asthma and COPD services /support**

17. What are the main barriers that hinder you from supporting or providing services to asthma and COPD patients?

Prompt: Can you use an example to explain this?

**E) Further additions/ comments**

18. Is there anything else you would like to share with me regarding your role in optimising asthma and COPD management?
